# The Role of Nanoparticles in Wine Science: Innovations and Applications

**DOI:** 10.3390/nano15030175

**Published:** 2025-01-23

**Authors:** Agnieszka Mierczynska-Vasilev

**Affiliations:** 1 The Australian Wine Research Institute, Waite Precinct, Hartley Grove cnr Paratoo Road, Adelaide, SA 5064, Australia; agnieszka.mierczynska-vasilev@awri.com.au; 2 College of Medicine and Public Health, Flinders University, Sturt Road, Adelaide, SA 5042, Australia

**Keywords:** wine, nanoparticles, oenological sensor, antimicrobial agents

## Abstract

Viticulture, the science of growing, cultivating, and harvesting grapes, and enology, the art and science of making wine, are rapidly evolving through innovative approaches aimed at improving the quality and efficiency of grape and wine production. This review explores the emerging use of nanoparticles, in particular gold, silver, and magnetic nanoparticles, to improve the quality, safety, and sustainability of both grape growing and winemaking processes. The unique properties of these nanoparticles, such as their small size, high surface area, and distinct chemical properties, enable them to address key challenges within the industry. In viticulture, nanoparticles have shown potential in protecting vines from pathogens, optimizing grape yield, and improving quality. In enology, nanoparticles are making a significant contribution to microbial control, reducing spoilage and refining wine analysis techniques, leading to improved product quality and safety. This review also highlights the synergy between different types of nanoparticles and their diverse applications, from microbial control in wine production to their use in innovative packaging solutions. In addition, nanoparticles have the potential to reduce dependence on agrochemicals and improve the sustainability of wine production, which is a promising avenue for future research. However, the integration of nanoparticles in viticulture and enology also poses regulatory and safety challenges, including the potential for nanoparticles to leach into wine products. Further research and regulatory advances are essential to ensure the safe and effective use of these technologies in winemaking. Overall, nanoparticles offer significant benefits to the wine industry, driving improvements in efficiency, sustainability, and quality.

## 1. Introduction

Nanoscience is a key area of modern science that focuses on the study of nanoparticles. These particles, typically between 1 and 100 nanometers in at least one dimension, exhibit remarkable electrical, optical, thermal, catalytic, and magnetic properties as a result of their tiny size [1,2,3]. Nanoparticles provide a practical alternative to expensive analytical equipment, as they are affordable to produce and can be recycled [4]. Nanotechnology, a field of research that emerged in the 20th century, has made remarkable progress since Nobel laureate Richard Feynman first introduced the concept in his influential 1959 lecture, “There is Plenty of Room at the Bottom” [5]. The focus of the discipline is on the application of nanoscience to create innovative products and technologies. Although relatively new, nanotechnology has already found extensive applications or thorough investigation in diverse fields such as biomedicine [6,7], environmental science [8,9], electronics [10,11], energy harvesting [12,13], mechanical industries [14,15], food and agriculture [16,17,18], and cosmetics [19,20]. In addition, with the recent progress in information and communication technologies (ICTs), nanotechnology is emerging as the hallmark of the industrial revolution of the 21st century, demonstrating its transformative potential across a range of sectors [21,22].

Focusing on the food industry specifically, the unique properties of nanoparticles offer unparalleled advantages in improving wine production and analysis due to their small size and distinct properties. Winemaking is an ancient practice with techniques that have been passed down through generations, but that does not mean it is stuck in the past. Modern challenges are being met head-on by winemakers who are producing creative solutions. Climate variability, sustainability concerns, and evolving consumer preferences are transforming the wine industry. New approaches are being sought that combine traditional methods with cutting-edge technology [23]. Nanotechnology has the potential to minimize reliance on the excessive use of agrochemicals, thus promoting sustainable agricultural practices [24]. In viticulture, nanoparticles show great promise in protecting grapevines from pathogens and optimizing grape yield and quality. In winemaking, nanoparticles can significantly contribute to controlling the growth of spoilage microorganisms and reducing undesirable compounds, resulting in improved wine quality and safety [25,26]. Gold and silver nanoparticles, known for their antimicrobial properties, are used in water purification and the development of antimicrobial packaging materials [27,28]. Additionally, the antibacterial activity of silver nanoparticles has been well documented in various matrices, including in biomedical applications, where they are utilized in wound dressings and coatings for medical devices due to their ability to combat multidrug-resistant bacteria [29]. In environmental settings, gold and silver nanoparticles have been studied for their ability to deactivate harmful bacteria and viruses in water treatment processes, effectively reducing pathogen load and ensuring cleaner, safer drinking water [30].

Magnetic nanoparticles can also help to remove yeast sediment from sparkling wines, facilitating clarification processes [31]. Their response to external magnetic fields makes them an excellent tool for separation and purification processes in the food and beverage industry. Nanoparticles are also revolutionizing wine analysis through improved sensor technologies. Their integration increases sensitivity, accuracy, and selectivity while reducing response times and sample pretreatment requirements. A variety of nanomaterials, including carbon nanodots [32,33], nanotubes [34,35,36], gold [37,38,39,40], silver [41,42], zinc oxide [41], and iron oxide nanoparticles [43,44,45], allow the accurate detection and quantification of wine components, helping to refine and improve analytical methods.

Beyond the food industry, nanoparticles are being explored for their diverse applications in other sectors. The antimicrobial properties of nanoparticles have been explored across various materials, including textiles, paints, and coatings, to improve surface protection against bacterial growth [46]. For example, titanium dioxide nanoparticles have been incorporated into self-cleaning surfaces to inhibit microbial contamination [47], while copper nanoparticles have been employed in agriculture to prevent the growth of plant pathogens [48]. Similarly, in pharmaceuticals, gold nanoparticles have been shown to exhibit antibacterial properties and have potential as drug delivery carriers, further demonstrating their versatility in both industrial and biomedical fields [49]. In food and beverage applications, these advancements have the potential to improve hygiene standards and contribute to the development of safe, high-quality products [46].

In summary, incorporating nanoparticles into viticulture and enology has the potential to revolutionize the industry. These advanced materials can enhance vine protection and improve wine analysis, delivering numerous benefits. By boosting quality, safety, and efficiency, nanoparticles are set to play a crucial role in shaping a more sustainable and forward-looking future for winemaking. This review hypothesizes that the integration of gold, silver, and magnetic nanoparticles can transform current grape growing and winemaking practices, leading to a more sustainable and innovative future for the industry.

Although there is growing interest in the use of nanoparticles in food production, there is a significant gap in the literature regarding their full integration into vineyard management and winemaking processes. The objective of this review is to explore the diverse types of nanoparticles, specifically gold, silver, and magnetic nanoparticles, and their potential functions in vineyard management, fermentation, wine aging, and quality enhancement. The purpose of this review is to examine the existing knowledge gap by synthesizing the latest research on the specific applications of these nanoparticles, evaluating their benefits and challenges, and charting a pathway for their successful incorporation into sustainable winemaking practices. By providing an in-depth analysis of the scientific advancements and opportunities, this review seeks to pave the way for future innovations in the field.

For this review, a systematic literature search was conducted in major academic databases, including Scopus, Web of Science, and PubMed. The included studies were selected based on their relevance to the use of nanoparticles in winemaking and viticulture, with a specific focus on gold, silver, and magnetic nanoparticles. Only peer-reviewed articles published in reputable journals were considered, and studies were selected based on their contribution to advancing the understanding of nanoparticle technology in viticulture and enology.

## 2. Oenological Sensors

Modern sensing technologies can be used to accurately identify multiple compounds within the wine matrix. The use of nanomaterials is key to enhancing sensor performance and improving the surface area of the electrode. This, in turn, leads to greater detection sensitivity and selectivity, as demonstrated by Barroso in 2011 [50]. Sensitivity is a critical requirement in sensor applications. In general, device sensitivity decreases with increasing film thickness, as observed in research by Riul et al. in 2003 [51]. Achieving high sensitivity requires using electrodes with interdigitated patterns coated with nanostructured thin films, each layer about two nanometers thick. These nanostructured coatings can detect even the slightest variations in electrical conductivity and dielectric properties within the materials of each sensor when they come into contact with a solution, as demonstrated by Riul et al. in 2004 [52]. Gold, silver, and magnetic nanoparticle-based sensing in enology has potential to improve wine analysis, quality control, and the detection of specific wine-related compounds. Each type of nanoparticle brings its own benefits and applications to the wine industry.

### 2.1. Gold Nanoparticle (AuNP)-Based Sensing

#### 2.1.1. Gold Nanoparticles in Wine Analysis

Nanoparticles, particularly gold nanoparticles (AuNPs), have emerged as powerful tools in wine analysis and quality control due to their distinctive characteristics and versatile applications. One of the most notable characteristics of AuNPs is their surface plasmon resonance (SPR) property, which induces changes in their absorption and scattering behavior when interacting with analytes present in wine samples. Taking advantage of this phenomenon, AuNP-based sensing techniques provide a sensitive and accurate way to detect and quantify specific compounds in wine, facilitating comprehensive analysis and quality assessment.

AuNPs can also be functionalized with various molecules such as ligands, antibodies, or aptamers, allowing selective interaction with target wine components. The functionalization process may induce changes in the optical properties of the AuNPs when they are bound to the compounds of interest, allowing them to be efficiently detected and measured [53]. In addition to basic compositional analysis, AuNP sensors show promise in flavor and taste profiling, enabling the identification and quantitation of key sensory characteristics in wine by detecting key compounds responsible for these properties. They can identify and quantify compounds that influence wine’s aroma and taste, such as phenolic acids, flavonols, and other polyphenolic compounds, which are vital to the organoleptic properties of wine [54,55]. These sensors can also play a critical role in quality control by rapidly detecting contaminants, off-flavors, or undesirable compounds that can compromise the integrity and taste of wine products [54]. AuNP-based sensors may also help authenticate wines by targeting specific markers unique to certain regions or varietals, helping to combat counterfeiting. In addition, AuNP-based sensors can be used to monitor fermentation by tracking the levels of important fermentation markers, helping winemakers optimize fermentation conditions to achieve desired wine properties.

#### 2.1.2. Advances in AuNP-Based Biosensors

Overall, the integration of AuNP-based sensing technologies offers a comprehensive approach to improving wine analysis, quality control, and production optimization [54,55] and also extends into the field of biosensors. For instance, the immobilization of enzymes such as tyrosinase on glassy carbon electrodes coated with electrodeposited gold nanoparticles has facilitated the development of biosensors with remarkable analytical performance, as demonstrated by Sanz et al. (2005) [56]. These biosensors exhibit excellent repeatability of measurements, do not require cleaning pretreatment, and show extended durability and consistent performance in their assembly.

Moreover, they demonstrate high sensitivity for the amperometric analysis of phenolic compounds, similar to the analytical characteristics reported in the literature for tyrosinase bioelectrodes. The rapid and straightforward application of these biosensors, called TyrnAu GCEs, involves simply introducing an aliquot of wine into the electrochemical cell to measure the levels of phenolic compounds in wines. Thus, AuNP-based biosensors offer potential for improving wine authentication, fermentation monitoring, and quality control by bridging the gap between advanced sensing technologies and practical applications in wine analysis.

Curulli et al. (2012) [37] contributed to the advancement of AuNP-based sensing techniques by developing a chitosan–AuNP-based sensor for determining the polyphenol content. Their method enhances the accuracy and reliability of polyphenol measurements by using nanomaterials that reduce interference from molecules such as ascorbic acid. Importantly, this approach provides a viable alternative to complex chromatographic methods that require multiple preparation steps and avoids the need for complex sample pretreatment. The proposed method is straightforward, quick, and cost-effective, making it particularly attractive for routine analysis in wine production.

#### 2.1.3. Innovations in Electrochemical Platforms

Sánchez-Obrero et al. (2012) [38] expanded the application of AuNP-based biosensors in wine analysis by developing a composite electrode for the detection of polyphenolic compounds, specifically catechol and phenol. By cross-linking the tyrosinase enzyme using glutaraldehyde, they constructed a biosensor with excellent applicability in both batch and flow injection analysis (FIA) systems, exhibiting remarkable reproducibility and stability. Operating at an ultra-low potential, the biosensor demonstrated a linear response, with detection limits of ~8 × 10^−7^ M for phenol and ~6 × 10^−7^ M for catechol.

Importantly, the low operating potential minimizes the influence of interference from species such as ascorbic acid, ensuring accurate and reliable identification of polyphenols in must and wine samples. The efficacy of the biosensor was further validated through a comparison with the Folin–Ciocalteu reference procedure, showing a strong correlation with the obtained results. These results highlight the potential of this biosensor as a valuable approach for the fast and automated assessment of ‘total phenolics’ and ‘antioxidant capacity’ in both must and commercial wines.

Lanzellotto et al. (2014) [57] contributed to the advancement of electrochemical biosensing platforms by introducing an innovative method combining gold nanoparticles and fullerenes, which possess unique electrochemical properties. Gold nanoparticles have attracted considerable attention for their attractive electrocatalytic behavior, which has been the driving force behind numerous sensing applications in recent years. Conversely, although their full potential in biosensing remains largely untapped, fullerenes and their derivatives represent a promising class of electroactive compounds.

In their study, Lanzellotto et al. (2014) [57] developed a biosensor based on laccase using a multilayer structure comprising AuNPs, fullerenes, and Trametes versicolor Laccase (TvL), meticulously assembled layer by layer on a gold electrode surface. This biosensor exhibited rapid amperometric responses to gallic acid, a recognized standard for polyphenol analysis in wine. It showed a low detection limit (LDL) of 0.006 mmol L^−1^. The enzymatic sensor detected polyphenols in buffers and wine samples, showing it could be a valuable tool for rapid and accurate wine analysis.

#### 2.1.4. Emerging Applications and Future Prospects

##### Future Prospects in Detecting Polyphenols and Sulfur Dioxide with Inkjet-Printed Electrodes

Schneider et al. [58] (2014) addressed the challenge of detecting simultaneously polyphenols and sulfur dioxide by employing inert electrodes in cyclic voltammetry. Traditional methods have encountered difficulties due to the proximity of the oxidation potentials of these compounds. Schneider et al. [58] presented a solution with inkjet-printed electrodes. These electrodes generate a voltammetric signal that both inhibits polyphenol oxidation and shows a linear correlation between sulfur dioxide concentration and the oxidation peak height.

Among the different types of printed working electrodes, those composed of gold nanoparticles combined with silver exhibited the highest sensitivity to sulfur dioxide. The cost-effective fabrication of these sensor elements, coupled with the rapid detection of sulfur dioxide through cyclic voltammetry, makes this technique particularly promising for application in the wine industry. The use of different working electrode types, in particular gold nanoparticle/silver combinations, demonstrates the versatility and effectiveness of this approach. Inkjet-printed electrodes are a valuable tool for improving quality control and monitoring sulfur dioxide levels in wine production due to their combination of accuracy, cost-effectiveness, and rapid detection.

##### Advancements in Electrode Modification with Functionalized AuNPs for Wine Analysis

In 2015, Medina-Plaza et al. [39] developed a method to chemically modify electrodes using gold nanoparticles functionalized through the Langmuir–Blodgett (LB) process. The resulting SDODAuNP-LB electrodes demonstrated excellent surface characteristics and proved highly adept at detecting organic acids and phenolics, crucial components that influence the acidity and antioxidant properties of grapes, musts, and wines. By employing the Langmuir–Blodgett method on these amphiphilic nanoparticles, sensors with an increased surface area-to-volume ratio and enhanced electrocatalytic activity were developed, resulting in a significant boost in peak currents. Notably, the detection limits achieved with SDOD-AuNP-LB sensors were considerably lower than those observed with ITO glass electrodes, falling in the order of 10^6^ mol L^−1^. Additionally, these sensors demonstrated the ability to discriminate dominant species based on pKa and pH, allowing the combined detection of organic acids and phenolic compounds without interference within the pH and concentration ranges typically found in wines and musts. Modified SDOD-Au-NP-LB electrodes show great potential for use in multi-sensor systems for comprehensive must analysis. This approach demonstrates the value of gold nanoparticle-based electrode modifications in improving analytical methodologies and understanding of key components in winemaking.

##### Expanding the Use of AuNPs and Reduced Graphene Oxide for Sensory Applications in Wine Analysis

Jiang et al. [40] pioneered the development of a platform combining gold nanoparticles and reduced graphene oxide (AuNPs-rGO) for detecting ochratoxin A (OTA), offering an exceptional signal amplification framework for an impedance aptasensor. By incorporating AuNPs into graphene, an abundance of binding sites for reporter DNA were provided, enabling each rGO sheet and AuNP to accommodate hundreds of reporter DNA molecules. Consequently, even minute residual aptamer amounts exhibited remarkable efficiency in binding a substantial number of negatively charged phosphate backbones. This synergistic effect led to a significantly enhanced Rct (charge transfer resistance) signal for the redox probes, surpassing that obtained with aptamers alone without the functionalized AuNPs-rGO platform. This approach achieved an impressive limit of detection of 0.74 pM, nearly 200 times better than the detection limits of many other existing impedimetric aptasensors. Furthermore, the versatility of functionalizing AuNPs and/or rGO with different signal probes and reporter DNA opens up possibilities for using this sensor design to amplify signals in aptamer-based detection across various molecules and analytical techniques. This breakthrough shows how nanomaterials can improve biosensing, paving the way for new analytical methods in wine analysis.

##### Future Implications of Surface Plasmon Resonance Biosensors for Contaminant Detection in Wine

Building on these advances, two years later, Karczmarczyk et al. [59] developed a rapid and highly sensitive sensor for detecting ochratoxin A (OTA) in red wine, using gold nanoparticle-enhanced surface plasmon resonance. This sensor was capable of detecting OTA at levels as low as 0.75 ng mL^−1^, and with the integration of AuNPs as signal enhancers, the detection limit was significantly improved by more than an order of magnitude, reaching 0.068 ng mL^−1^. In addition, this work investigated a complex relationship between AuNP size and recognition component affinity and elucidated how this interplay affected the efficacy of the AuNP-based signal enhancement strategy. In particular, polyphenolic compounds in the wine samples posed a significant challenge by interfering with the binding interactions on the sensor surface. To overcome this, a simple pretreatment using the binding agent poly(vinylpyrrolidone) (PVP) was successfully implemented, which effectively mitigated the interference and improved the performance of the biosensor in wine sample analysis. This work highlights the continuing refinement and adaptation of biosensing technologies, particularly in the wine industry, to achieve greater sensitivity, accuracy, and reliability in detecting contaminants and ensuring product quality and safety.

##### Emerging Potential of Disposable Electrochemical Immunosensors for Wine Quality and Safety

Continuing the trajectory of innovation in biosensing technologies, Borisova et al. [60] introduced a groundbreaking approach with an innovative, disposable electrochemical immunosensor. This sensor used screen-printed carbon electrodes, modified with a hybrid nanomaterial comprising gold nanoparticles and graphene. These modified electrodes were further functionalized with a targeted anti-Brett polyclonal antibody. The electrode with nanostructured design developed by Borisova and her team exhibited remarkable sensitivity in detecting yeast at very low concentrations, both in controlled solutions and commercial red wine samples. The impressive analytical performance achieved with this immunosensor highlights the potential of screen-printed electrodes enhanced by this novel nanohybrid to serve as the foundation for highly responsive, reliable, and single-use electrochemical devices tailored for food analysis. Biosensors, particularly those focused on food safety and quality assurance, hold great promise in improving detection capabilities and ensuring consumer health.

#### 2.1.5. Conclusions

AuNPs are widely recognized as powerful tools in wine analysis due to their surface plasmon resonance property, which allows the precise detection and quantification of compounds. These AuNP sensors are functionalized with specific molecules, such as antibodies or aptamers, which enable selective interactions with target compounds, including phenolic acids and polyphenols that are crucial to wine’s taste and aroma. With their high sensitivity, these sensors are suitable for various applications, including flavor profiling, wine authentication, and quality control. They are particularly effective in monitoring fermentation processes and detecting contaminants or off-flavors. However, there are some drawbacks, such as the potential interference from compounds like polyphenols in complex wine matrices and the high costs associated with the synthesis of gold nanoparticles and the development of sensors.

Advances in AuNP-based biosensors have significantly enhanced analytical performance in wine analysis. The integration of enzymes, such as tyrosinase, onto gold nanoparticle electrodes has improved the biosensors’ sensitivity, repeatability, and durability. These biosensors are particularly effective for analyzing phenolic compounds in wine, offering a more efficient alternative to traditional methods that often require complex pretreatment. The advantages of AuNP-based biosensors include high repeatability and durability, their effectiveness in phenolic compound analysis, and their simplicity and cost-effectiveness for routine wine production analysis. However, a key drawback is that these sensors are generally limited to specific wine compounds like phenolics, which may restrict their ability to detect a broader range of compounds.

Innovations in electrochemical platforms utilizing AuNP-based biosensors, such as those developed by Sánchez-Obrero et al. [38], demonstrate reproducibility and low operating potential, which help minimize interference from compounds like ascorbic acid. These sensors are designed for fast and automated detection of polyphenolic compounds and antioxidant capacity in wines, enhancing the efficiency of wine analysis. The advantages of these platforms include low operating potential that reduces interference, reliability for both batch and flow injection analysis, and fast, automated detection methods. However, the drawbacks include the limitation of detection to specific polyphenolic compounds and the need for sophisticated equipment and setup.

Emerging applications of AuNPs in wine analysis include the use of inkjet-printed electrodes for sulfur dioxide detection, which enhances quality control by addressing previous challenges in simultaneously detecting polyphenols and sulfur dioxide. The advantages of this approach include cost-effective and rapid detection methods, versatility in detecting multiple compounds such as sulfur dioxide, and its potential for improving wine quality control. However, the drawbacks include the need for advanced printing technologies to produce the electrodes and potential challenges in scaling up production for widespread use.

### 2.2. Silver Nanoparticle (AgNP)-Based Sensing

Silver nanoparticles (AgNPs) have become versatile tools in wine production and its quality management. With their natural antimicrobial properties, AgNPs can be used for detecting and controlling spoilage microorganisms in wine, ensuring microbiological stability and extending shelf life. In addition, AgNP-based sensors offer a reliable platform for detecting mycotoxins, heavy metals, and pesticides, thereby enhancing wine safety and regulatory compliance. Beyond contamination detection, these sensors can also be used for environmental monitoring within vineyards, enabling assessment of soil and water quality to support sustainable wine production practices and maintain vine health. In addition, AgNP-based sensors are used in packaging analysis to evaluate the influence of packaging materials on wine quality, in particular to detect compounds that can leach from packaging into wine, providing valuable insight into packaging integrity and its influence on wine characteristics. AgNPs are used extensively in wine production to ensure quality, safety, and sustainability.

In 2012, Chawla et al. [41] demonstrated that AgNPs can enhance biosensor performance for measuring phenolic compounds in wine. The researchers achieved this by covalently immobilizing laccase on a nanocomposite film of AgNPs and electrochemically deposited zinc oxide nanoparticles (ZnONPs) on a gold (Au) electrode. This approach significantly improved analytical performance, characterized by very low operating potential (0.22 V), fast response (8 s), increased sensitivity (0.71 µA µM^−1^ cm^−2^), and impressive storage stability of up to five months. The use of AgNP/ZnONP composite films in the design of biosensors demonstrated their effectiveness in the measurement of phenol content and showed their ability to enhance the performance of various other biosensors. The study highlights the diverse applications of AgNPs in biosensing and emphasizes their importance in providing accurate and reliable analytical measurements in the wine industry.

Continuing the exploration of silver nanoparticle (AgNP)-based sensing applications in enology, Karabiberoglu et al. [42] presented a novel approach using a poly(thiophene)-modified glassy carbon electrode (GCE) decorated with AgNPs for the determination of caffeic acid. This electrode showed a linear relationship between peak current and concentration over a range of 1.00 × 10^−8^ to 4.83 × 10^−6^ M, with a detection limit of 5.3 × 10^−9^ M, and significantly enhanced electrocatalytic activity tailored explicitly to the oxidation of caffeic acid. Furthermore, the modified electrode successfully determined the concentration of caffeic acid in red wine samples, enhancing quality control measures during winemaking. Overall, winemakers and researchers have a powerful potential set of tools to gain comprehensive insight into wine composition and quality by integrating AgNP-based sensing techniques into the various stages of wine production, from grape growing to packaging.

#### Conclusions

AgNPs have proven to be versatile tools in wine production, offering a range of applications that enhance wine quality, safety, and sustainability. Their natural antimicrobial properties help detect and control spoilage microorganisms, thereby ensuring microbiological stability and extending the shelf life of wine. AgNP-based sensors also provide reliable platforms for detecting mycotoxins, heavy metals, and pesticides, ensuring wine safety and regulatory compliance.

In vineyards, these sensors can monitor soil and water quality, supporting sustainable practices and maintaining vine health. Additionally, AgNP sensors are used in packaging analysis to evaluate the impact of materials on wine quality, detecting any harmful compounds that might leach into the wine. Notable studies, such as those by Chawla et al. (2012) and Karabiberoglu et al. (2012) [41,42], demonstrate the enhanced performance of AgNP-based sensors in detecting phenolic compounds and caffeic acid, respectively, contributing to more accurate quality control in winemaking.

Despite their advantages, there are concerns regarding the potential environmental and health risks of AgNPs, as well as the cost and specialized equipment required for their use, which may limit their adoption, especially among small-scale producers. Nonetheless, AgNP-based sensing technologies hold great promise in improving the overall quality and sustainability of wine production, provided that their potential risks and costs are carefully managed.

### 2.3. Magnetic Nanoparticle (MNP)-Based Sensing

Magnetic nanoparticles (MNPs) have become essential tools in many aspects of wine production, providing unique capabilities in selective detection, quality control, fermentation monitoring, environmental assessment, wine maturation, and research and development. Functionalization tailors MNPs to selectively interact with specific wine components or contaminants, using their magnetic properties to assist in the separation and concentration of target analytes within intricate wine matrices. In quality control, MNP sensors can be used to detect unwanted compounds and off-flavors, providing fast, accurate, and selective analysis to ensure wine quality standards are met. They can also be used to monitor fermentation markers such as sugars and organic acids, enabling winemakers to optimize the fermentation process for desired wine characteristics. In vineyards, MNP-based sensors can play a key role in assessing environmental factors such as heavy metals, pesticides, and contaminants. In addition, MNP-based sensors can provide insight into the aging process of wines by monitoring changes in wine composition over time. These sensors have the potential to drive advances in winemaking techniques and product innovation and support wine research efforts through a deeper understanding of wine chemistry and sensory attributes. Essentially, MNP-based sensing technology is transforming winemaking from vine to bottle, driving continuous improvement and innovation in the wine industry.

Furthermore, the application of magnetic nanoparticles (MNPs) extends beyond traditional winemaking processes, addressing critical aspects such as the detection of harmful contaminants. Fernández-Baldo et al. [43] demonstrated the feasibility of monitoring ochratoxin A (OTA), a fungal metabolite with carcinogenic and nephrotoxic properties, using an electrochemical method combined with modified MNPs. This approach, which offers advantages such as economic viability, wide linear range, reproducibility, accuracy, and an excellent detection limit, involves a specific antigen–antibody reaction on modified MNPs. Successfully applied to various red grape varieties, this method holds promise as an analytical tool for direct OTA detection in real samples, particularly as tolerance levels for OTA in fruit are established. Building on this advancement, Zamfir et al. [45] developed a label-free immunosensor for OTA detection using MNPs. Their approach involved modifying a gold electrode with bovine serum albumin conjugate to prevent nonspecific OTA binding, followed by linking OTA antibodies to MNPs using carbodiimide chemistry. The complementary nature of these studies highlights the versatility and efficacy of MNP-based sensing technologies in addressing critical challenges in wine safety and quality assurance.

Expanding the repertoire of magnetic nanoparticle (MNP)-based sensing in wine analysis, Rawal et al. [44] introduced an innovative approach by developing an amperometric sulfite biosensor. Their method involved covalently attaching sulfite oxidase to gold-coated MNPs using N-ethyl-N-(3-dimethyl aminopropyl) carbodiimide (EDC) and N-hydroxysuccinimide (NHS) chemistry. This biosensor demonstrated remarkable accuracy in measuring sulfite levels in both red and white wines. By leveraging a modified electrode, Rawal et al. achieved improved analytical performance, characterized by a short response time (2 s), a lower detection limit of 0.15 M, extended storage stability (up to 120 days), a wider linear range (0.5–1000), and minimal interference from various substances compared to previous biosensors. These results highlight the potential of the composite to enhance the analytical performance of other biosensing devices. Through continuous innovation and refinement, MNP-based biosensors show ongoing potential to advance the capabilities of wine analysis.

#### Conclusions

MNPs are crucial tools in wine production, offering applications in quality control, fermentation monitoring, environmental assessment, and wine maturation. Their functionalization enables selective interaction with specific wine components, facilitating the separation of target analytes. MNP-based sensors detect off-flavors and unwanted compounds and monitor fermentation markers, enhancing wine quality. They also assess environmental factors like pesticides and contaminants in vineyards and provide insights into wine aging.

Studies demonstrate MNPs’ effectiveness, such as OTA and sulfites. Fernández-Baldo et al. (2012) [43] used MNPs for OTA detection, providing economic viability, accuracy, and a wide linear range. Zamfir et al. (2013) [45] developed a label-free immunosensor for OTA with high sensitivity. Rawal et al. (2014) [44] introduced a sulfite biosensor with a short response time, low detection limit, and extended stability.

MNP-based sensors offer the advantages of high sensitivity, versatility, and rapid, accurate results throughout various stages of wine production, helping to ensure wine quality, safety, and sustainability. However, the technology can be costly and complex, requiring specialized equipment, which may limit its adoption, particularly among small producers. Additionally, interference from other substances in the wine matrix can present challenges to the technology’s effectiveness.

## 3. Nanoparticles for Removing and Reducing Undesirable Compounds in Wine

In the wine industry, nanoparticles can be used in the selective removal or reduction of specific compounds that affect wine quality and sensory properties. These nanoscale materials offer unique properties that make it possible to target and remove undesirable substances while preserving desired wine components. In one example, gold nanoparticles (approximately 20 nm in size) were synthesized by Liu et al. [61] using sonoelectrochemical methods in a 0.1 M NaCl solution without the use of any stabilizers. These prepared AuNPs served as efficient catalysts for the degradation of 2000 ppm aldehyde in 40% ethanol–water mixtures. After a ten-day test, approximately 50% of the aldehyde was eliminated from alcohol solutions containing 25 ppm AuNPs with gentle stirring. This method can also be applied to commercial wines to effectively eliminate unwanted aldehydes.

Building on the catalytic potential of gold nanoparticles in wine, further advancements were made by Yu et al. [62] in synthesizing size-adjustable AuNPs on chitosan in aqueous solutions. Initially, gold substrates were subjected to 200 cycles in deoxygenated aqueous solutions containing 0.1 N NaCl and 1 g/L chitosan, followed by exposure to UV light of varying wavelengths to create size-controllable AuNPs on chitosan. Using longer-wavelength UV light produced larger nanoparticles, with sizes in the range of 10 to 50 nm. The presence of these AuNPs on chitosan significantly enhanced the decomposition of acetaldehyde in wines, exhibiting an approximate 190% increase in effectiveness compared to pure AuNPs. In addition to showcasing the tunability of AuNPs, this approach underscores their potential for catalytic applications in the wine industry.

Cheng et al. [63] demonstrated the production of size-controllable AuNPs using sonoelectrochemical methods, with natural chitosan playing a pivotal role. By generating positively charged gold complexes in 0.1 N NaCl through oxidation–reduction cycles (ORC) on a gold substrate, they synthesized AuNPs with diameters ranging from 10 to 80 nm by adjusting chitosan concentrations during sonoelectrochemical reduction. The optimal chitosan-to-gold ratio for producing the smallest AuNPs was determined to be approximately 3. The catalytic activity of these size-controllable AuNPs was evaluated in the decomposition of acetaldehyde, revealing significant activity in 10 nm AuNPs compared to larger 80 nm counterparts.

Ou et al. [64] further refined the synthesis process by developing a novel sonoelectrochemical approach to produce precursors of microsized gold sheets, using hexadecyltrimethylammonium bromide (CTAB) as a capping agent. The release of AuNPs could be precisely controlled by adjusting the pH of the solution after CTAB removal using these microsized gold sheets, which were then exposed to sunlight lamps. Laying the groundwork for the exploration of various biomedical applications, these controllably released AuNPs proved effective in eliminating toxic acetaldehyde from ethanol solutions. This approach highlights the potential of AuNPs in wine-related catalysis and paves the way for broader applications in biomedical fields.

Berovic et al. [31] developed an efficient magnetic separation method for isolating yeast cells in sparkling wine. This is a promising alternative to conventional labor-intensive methods. By leveraging superparamagnetic iron oxide maghemite nanoparticles coated with silica and treated with (aminoethylamino) propylmethyldimethoxysilane (APMS), they rendered the yeast cells responsive to a magnetic field. Nanoparticles, positively charged by APMS, were electrostatically attracted to the negatively charged cell surfaces, enabling efficient separation. Through meticulous experimentation, an optimal nanoparticle-to-yeast mass ratio was determined at 1:10. Microscopic examination revealed sustained attachment of magnetic nanoparticles to microbial cell surfaces post-fermentation, with no detrimental effects on cell metabolism observed in chemical analyses. Sensory evaluation of the resulting sparkling wine confirmed these findings, with no adverse effects detected, confirming the safety and efficacy of the magnetic separation approach. In particular, the magnetic separation, which takes approximately 15 min using a relatively weak magnetic field gradient, contrasts sharply with the traditional method, which involves lengthy manual or pallet-based rotation for up to 60 days. Traditionally, this tedious process involves two distinct steps: riddling and disgorging, which are time-consuming and costly [65]. Using innovative techniques based on nanoparticles, Berovic et al. [31] offer a streamlined, cost-effective solution for enhancing the efficiency of yeast separation in sparkling wine production, marking a significant advancement in this field.

Building on the use of paramagnetic nanoparticles in wine production, Bettini et al. [66] synthesized paramagnetic iron oxide nanoparticles encapsulated with a silica shell, denoted as MNPs@SiO_2_, to enhance stability and enable effective binding with biogenic amines. Characterization via infrared and Raman spectroscopy highlighted magnetite and maghemite as the predominant components of the paramagnetic nanoparticles, emphasizing the crucial role of the surface layer in facilitating interactions with amines. The resultant magnetic complexes formed with biogenic amines exhibited swift and efficient removal under weak magnetic fields, demonstrating the potential of this novel approach for mitigating unwanted fermentation by-products in real commercial wine samples. Subsequent absorption spectra analyses confirmed the complete elimination of these compounds. By integrating paramagnetic nanoparticles and silica coating, Bettini et al. [66] propose a compelling avenue for addressing quality control challenges in wine production, offering a rapid and effective solution for the removal of undesirable fermentation by-products.

Dusak et al. [67] devised a novel method to magnetically extract magnetized lactic acid bacteria (LAB), specifically Oenococcus oeni, during malolactic fermentation (MLF). By attaching functionalized magnetic nanoparticles to bacterial surfaces in suspension and employing magnetoresponsive bacteria (MRB) during fermentation, the authors successfully used high-gradient magnetic separation (HGMS) to separate MRB from wine. This allowed for effective separation without compromising bacterial metabolism by precisely controlling the attachment of superparamagnetic, amino-functionalized, silica-coated maghemite nanoparticles to O. oeni cells. The application of MRBs during MLF, followed by their efficient removal via HGMS, facilitated the cessation of fermentation while maintaining wine quality.

Liang et al. [68] investigated the potential of putative imprinted magnetic polymers (PIMPs) as a solution for reducing elevated levels of (3-isobutyl-2-methoxypyrazine) IBMP in Cabernet Sauvignon grape must. Through comparative analyses with non-imprinted magnetic polymers (NIMPs) and a commercially available polylactic acid (PLA)-based film added post-fermentation, these authors demonstrated the superior efficacy of PIMP treatments in mitigating ‘fresh green’ aroma nuances while preserving overall aroma profiles. Their findings revealed that the post-fermentation addition of magnetic polymers effectively removed up to 74% of the initial IBMP concentration, surpassing the capabilities of PLA-based interventions. Moreover, although slightly less effective in IBMP reduction (20–30%), the pre-fermentation addition of magnetic polymers exhibited a gentler impact on other wine volatiles and color parameters.

**Figure 1:**
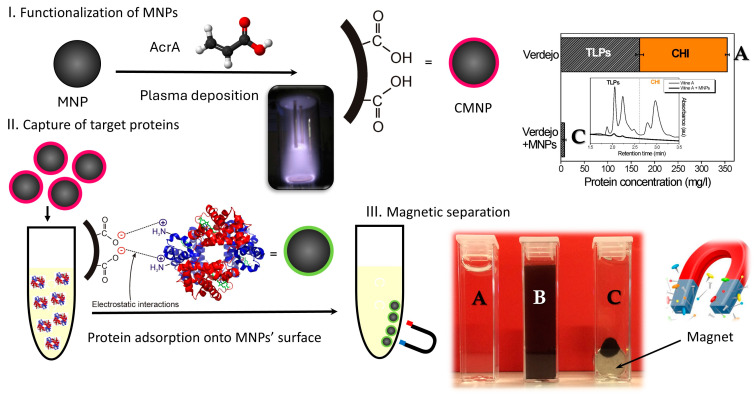
Schematic representation of the magnetic separation process for removing pathogenesis-related proteins from wine: (I) functionalization of MNPs using plasma deposition of acrylic acid; (II) capture of pathogenesis-related proteins, such as thaumatin-like proteins (TLPs) and chitinases (CHIs), from white wine, demonstrated using Verdejo wine as an example; (III) separation of pathogenesis-related proteins from wine by applying an external magnetic field. The photo with the red background illustrates **A** wine before treatment, **B** wine with dispersed MNPs, and **C** wine after the application of the magnetic field. Reprinted/adapted with permission from Ref. [69]. Copyright 2017, Elsevier.

In 2017, Mierczynska-Vasilev et al. [69] developed a new method for the rapid and selective elimination of pathogenesis-related proteins from white wines, as presented in Figure 1. In this work, pathogenesis-related proteins from various wines such as Verdejo, Riesling, Viognier, Semillon/Sauvignon Blanc, Vermentino, Sauvignon Blanc, and Chardonnay were efficiently captured and removed using magnetic nanoparticles coated with acrylic acid plasma. Further analysis demonstrated that the acrylic acid-coated magnetic nanoparticles effectively removed the proteins without significantly altering the phenolic content of the wines, making this technology a promising alternative to bentonite treatment, which is associated with economic losses and sensory impacts [70].

Expanding upon this technology [69], subsequent research endeavors aimed to optimize and diversify the functionality of magnetic nanoparticles (MNPs) [71]. Through the introduction of various functional groups, including amine (NH_2_), carboxyl (COOH), and oxazoline (POx), via allylamine, acrylic acid, and 2-methyl-2-oxazoline monomers, respectively, the research team developed plasma coatings enriched with distinct chemical functionalities. A comparison of these modified MNPs demonstrated their effectiveness in removing proteins from wine, with a performance ranking of COOH > POx > NH_2_. This optimization highlights the potential of MNPs for broader applications in various fields, including water treatment, biotechnology, and therapeutics, in addition to increasing their versatility for beverage production. The development offers a promising way to improve magnetic separation technologies and address current challenges in various industries. Subsequent research efforts have expanded the scope and utility of nanoengineered surfaces in wine production and quality improvement [72]. Through systematic investigations into the reusability of plasma-coated MNPs over multiple fining and regeneration cycles, the research team demonstrated the enduring efficacy of acrylic acid plasma-coated MNPs in eliminating pathogenesis-related proteins from white wines through successive adsorption–desorption processes. In addition, the preservation of essential wine constituents, including organic acids and phenolics, underlined the potential of this technology to maintain the original composition of the wine, thus preserving its quality throughout the fining process.

**Figure 2:**
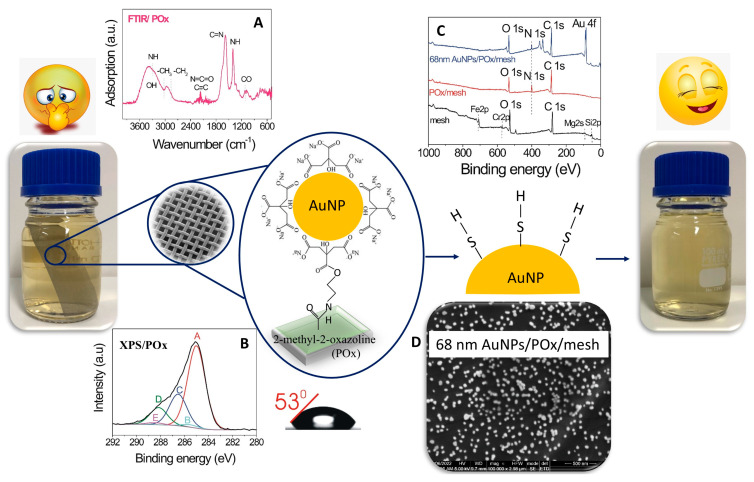
Schematic illustration of the generation of smart surfaces and their use in wine. (**A**–**C**) show the physicochemical properties of the coatings. (**A**) FTIR spectrum of 2-methyl-2-oxazoline coating deposited on KBr. (**B**) High-resolution C1s XPS spectrum of 2-methyl-2-oxazoline coating. (**C**) XPS survey spectra showing the surface chemical composition of uncoated mesh surfaces, mesh surfaces coated with POx, and mesh surfaces coated with POx and modified with 68 nm gold nanoparticles. (**D**) SEM image of 68 nm gold nanoparticles immobilized on POx deposited on a mesh surface. Reprinted/adapted with permission from Ref. [74]. Copyright 2023, Elsevier.

In a subsequent study by Mierczynska-Vasilev et al. [73], a novel approach for the targeted removal of major volatile sulfur compounds (VSCs) from wines was introduced, further exemplifying the versatility and applicability of nanoengineered surfaces in wine technology. This method combined chemical and structural surface modifications, leveraging a thin plasma polymer coating and immobilized AuNPs to selectively eliminate sulfidic off-flavors from wine. AuNPs were deliberately selected for their affinity to sulfhydryl compounds, which facilitates the formation of strong gold–sulfur bonds, ensuring the efficient removal of unwanted VSCs while preserving desirable flavor profiles. Figure 2 shows a schematic of this process.

Importantly, this treatment retained a positive/pleasant aroma of tropical thiol and ‘gunflint’ aromas in wine while removing unwanted off-flavors and maintaining optimal SO_2_ concentrations, which are critical for wine quality. With potential applications ranging from common filtration equipment and remediation devices to wine packaging materials, these nano-engineered surfaces offer a transformative approach to improving wine quality and sensory attributes.

### Conclusions

Nanoparticles offer promising solutions for removing and reducing undesirable compounds in wine, significantly improving wine quality and sensory properties. Gold nanoparticles (AuNPs) have demonstrated catalytic capabilities in eliminating aldehydes and acetaldehyde, with various synthesis methods, including sonoelectrochemical techniques, enhancing their effectiveness. For example, size-adjustable AuNPs synthesized on chitosan demonstrated improved acetaldehyde decomposition in wines, showcasing their catalytic potential. Additionally, magnetic separation methods have been developed, such as using superparamagnetic iron oxide nanoparticles, to efficiently isolate yeast cells in sparkling wine production, reducing time and cost compared to traditional methods. Magnetic nanoparticles have also been utilized to remove biogenic amines, fermentation by-products, and pathogenesis-related proteins from wine, offering a rapid and effective solution for quality control. Moreover, the application of imprinted magnetic polymers (PIMPs) has proven successful in reducing specific off-flavors, such as IBMP, while preserving overall aroma profiles. However, while these nanoparticle-based methods offer high efficiency, they also face challenges such as the complexity of synthesis processes, potential interference with other wine components, and the need for specialized equipment. Despite these limitations, nanoparticle-based techniques represent a transformative approach to wine production, providing significant improvements in quality, safety, and sustainability.

## 4. Silver Nanoparticles as an Antimicrobial Agent

Silver nanoparticles (AgNPs) have been shown to be a promising antimicrobial agent in enology, demonstrating a broad spectrum of antimicrobial activity. They are particularly effective against bacteria, yeasts, and molds that are commonly found in the winemaking process. Their mechanism of action disrupts cellular processes, damages membranes, and disrupts vital functions, resulting in microbial death or reduced viability. AgNPs can be incorporated into surface coatings in winemaking machinery, tanks, and barrels, where they release silver ions to create a hostile environment for microbes, protecting the integrity of the wine. AgNPs are also used in wine packaging materials, such as bottle caps, liners, and labels, to protect against microbial ingress during storage and preserve wine quality. In addition, their incorporation into nanofiltration processes facilitates the removal or inactivation of microorganisms in wine, contributing to both clarification and microbial stabilization. AgNPs offer the opportunity to reduce reliance on sulfur dioxide (SO_2_) as a preservative, which may be attractive to certain segments of wine producers. With their versatile antimicrobial properties, AgNPs are an asset in maintaining wine quality and meeting the evolving needs of the wine industry, as summarized in Table 1.

Research into alternative antimicrobial agents in wine production, such as the colloidal silver complex (CSC), is part of the industry’s search for effective and sustainable solutions. The potential of CSC as an alternative to sulfur dioxide in both red and white wine production was investigated in a 2012 study by Izquierdo-Cañas et al. [25]. Remarkably, the addition of 1 g/kg CSC to grapes effectively controlled acetic and lactic acid bacteria while still allowing the growth of Saccharomyces cerevisiae, similar to the performance of certain concentrations of sulfur dioxide. Importantly, the levels of silver detected in finished white and red wines remained well below legal limits, ensuring consumer safety. The use of CSC caused slight changes in the composition of the wine and led to a reduction in alcohol and acetaldehyde content compared to wines treated with sulfur dioxide. However, a significant disadvantage was the lack of antioxidant activity of CSC, which is a very important property of sulfur dioxide in ensuring wine stability. This highlights the wine community’s ongoing search for a balance between effective microbial control and the preservation of sensory and chemical properties that are integral to consumer enjoyment.

In further work on alternative antimicrobial agents in winemaking, Garde-Cerdan et al. [26] explored the antiseptic properties of colloidal silver (CAgC) in red wine production, either alone or in conjunction with sulfur dioxide (SO_2_). Their study, conducted two years after Izquierdo-Cañas et al.’s research, aimed to assess the efficacy of CAgC during vinification and storage. Four treatment groups were established, incorporating various combinations of SO_2_ and CAgC. Remarkably, all grape treatments effectively controlled yeast and lactic acid bacteria, with CAgC treatments demonstrating superior control over acetic acid bacteria. Despite its antimicrobial effectiveness, the addition of CAgC did not negatively impact fermentation, and the resulting wines exhibited similar physicochemical, aromatic, and sensory characteristics to the control, albeit with a slightly reduced alcohol content. However, bacterial control was not sustained during storage, although wines produced with CAgC displayed increased color intensity and lower concentrations of anthocyanins and total polyphenols post-stabilization. Encouragingly, the volatile and biogenic amine compositions remained unchanged, with no discernible negative effects on quality or sensory attributes. This highlights the potential of CAgC as an effective antiseptic agent in the production and storage of young wines, providing effective microbial control while maintaining favorable sensory characteristics.

The inhibitory potential of novel biocompatible silver nanoparticles in winemaking was demonstrated by García-Ruiz et al. [95] in 2015. Their study focused on two distinct solid nanoparticle formulations, PEG-Ag NPs and GSH-Ag NPs solution, each exhibiting favorable physicochemical properties for antimicrobial applications. PEG-Ag NPs displayed remarkable effectiveness against Gram-negative bacterial strains, while GSH-Ag NPs exhibited efficacy against both strains, notably targeting Oenococcus oeni among wine lactic acid bacteria (LAB). Additionally, these nanoparticles demonstrated antimicrobial activity comparable to potassium metabisulfite, a commonly used antimicrobial agent in winemaking, against LAB and acetic acid bacteria (AAB). This research highlights the potential of AgNPs to control microbial processes in winemaking, offering insights into the design and application of antimicrobial-specific silver nanoparticles tailored for oenological use.

The effectiveness of alternative antimicrobial agents in winemaking was further examined by Izquierdo-Cañas et al. [97] in 2018, focusing on the kaolin silver complex (KAgC) as a potential substitute for sulfur dioxide (SO2). Their study revealed that a 1 g/L concentration of KAgC effectively inhibited the growth of Brettanomyces bruxellensis and acetic acid bacteria, two common spoilage microorganisms in winemaking. KAgC treatment significantly reduced the populations of these microorganisms, with residual yeast levels detected after 24 days and acetic acid bacteria reduced to negligible levels within 72 h. Furthermore, KAgC exhibited comparable efficacy to chitosan in lowering microbial populations in naturally contaminated wines. Wines treated with KAgC also showed reduced concentrations of acetic acid and 4-ethylphenol (a spoilage compound produced by Brettanomyces yeast) while staying within legal silver content limits, emphasizing KAgC’s effectiveness and regulatory compliance in controlling spoilage microorganisms during winemaking.

The exploration of alternative antimicrobial agents in winemaking continued in 2019 with the work of Gil-Sánchez et al. [98], who examined the efficacy of two kinds of silver nanoparticles, namely, PEG-AgNPs 20 and GSH-AgNPs, coated with biocompatible materials. Their study involved evaluating the ability of these AgNPs to control microbial growth in wines while addressing health concerns associated with sulfur dioxide (SO_2_). Interestingly, both types of AgNPs underwent in vitro digestion, with GSH-Ag NPs maintaining their size and shape, indicating their potential to reach the intestine in nanoscale form. In contrast, PEG-AgNPs 20 exhibited some particle agglomeration during digestion. Furthermore, Caco-2 cell experiments revealed no intestinal epithelial toxicity associated with these AgNPs. These findings suggest a promising role for AgNPs as alternatives to SO_2_ in winemaking, offering broad-spectrum antimicrobial benefits.

### 4.1. Benefits and Considerations of Using AgNPs

The use of AgNPs in the production of wine has several important advantages. Firstly, it could reduce the dependence on traditional ingredients, which may assist in the adoption of more environmentally friendly winemaking practices [25]. Secondly, AgNPs can effectively control microbial spoilage, leading to more consistent flavor profiles and sensory characteristics [99]. The antibacterial qualities of silver may also contribute to extending the shelf life of wine by inhibiting spoilage during storage and maturation [99]. However, to ensure the satisfaction and health of consumers, it is important to ensure that the use of AgNPs in enology is in line with regulatory guidelines and safety standards [99]. To further enhance the potential of AgNPs in the wine industry, continued research should aim at optimizing their application in winemaking and investigating their long-term effects on wine quality [100].

The economic importance of using nanoparticles (NPs) in viticulture and enology is evident in several key areas. First, the enhanced efficiency provided by NPs in wine production processes, such as faster filtration, more effective microbial control, and improved wine analysis, translates to cost savings. For example, the ability of nanoparticles to aid in the clarification of wines or to prevent spoilage can reduce the need for traditional chemical additives, such as sulfur dioxide, which can be expensive and have regulatory restrictions [25]. Furthermore, the integration of NPs into packaging materials could prolong the shelf life of wines, minimizing losses due to spoilage and improving the marketability of wine products [98]. Nanotechnology also has the potential to improve the sustainability of winemaking by reducing the reliance on agrochemicals, which are costly and environmentally taxing [101]. By improving both the quality and safety of wine while promoting more sustainable practices, NPs can contribute to a more economically viable and future-proof wine industry [102].

However, the use of nanoparticles in food and beverages does come with potential risks, particularly regarding their toxicology. The small size and high surface area of nanoparticles enable them to interact with biological systems in ways that larger particles cannot, raising concerns about their safety when consumed [103]. Nanoparticles may accumulate in human tissues, and their long-term effects on health are still not fully understood [104]. Studies have shown that certain nanoparticles could exhibit toxicity, causing oxidative stress, inflammation, and even potential cellular damage [105]. This is particularly concerning when NPs are incorporated into consumables like wine, where they might directly interact with the human digestive system [103]. The possibility of nanoparticles leaching into the final product, either during wine production or storage, needs to be carefully monitored and regulated to ensure that their concentrations remain within safe limits [104]. Regulatory bodies such as the U.S. Food and Drug Administration (FDA) and the European Food Safety Authority (EFSA) have been working to develop guidelines for the safe use of nanoparticles in food and beverages, but significant research is still required to understand the full scope of their potential health risks [105]. Therefore, while the economic and technological benefits of NPs are significant, their safety must be rigorously assessed to avoid unintended negative consequences for consumer health [103,104,105].

### 4.2. Conclusions

AgNPs have gained recognition as effective antimicrobial agents in winemaking due to their ability to combat bacteria, yeasts, and molds. By disrupting microbial cellular processes and damaging membranes, AgNPs help prevent spoilage and preserve wine quality. They are incorporated into surface coatings of winemaking machinery, tanks, and packaging materials like bottle caps and labels, where they release silver ions to create an inhospitable environment for microbes. Additionally, AgNPs assist in nanofiltration to remove or inactivate microorganisms, reducing the need for traditional preservatives like sulfur dioxide (SO_2_). Studies have also explored alternative antimicrobial agents like colloidal silver complexes (CSCs) and kaolin silver complex (KAgC), which have shown similar efficacy to SO_2_ in controlling microbial growth while maintaining wine quality. Although AgNPs offer environmental and health benefits, their use must align with regulatory standards to ensure safety, and ongoing research is crucial to optimize their application and assess long-term effects on wine stability.

## 5. Nanoparticles Used in Wine: Challenges and Regulations

When considering the challenges and regulatory aspects related to nanoparticles in the wine industry, several important factors must be taken into account. The regulatory landscape for nanoparticles in food and beverages is multifaceted and continuously evolving. As the use of nanomaterials in the industry grows, regulatory bodies are working to develop clear guidelines that ensure consumer safety while enabling innovation. In many countries, nanoparticles are covered by existing regulations that apply to conventional food additives, but with additional scrutiny due to their unique properties and potential risks. Regulators require rigorous risk assessments to evaluate the safety of nanoparticles, focusing on toxicity, exposure levels, and potential health effects [106].

In addition, certain jurisdictions impose labeling requirements for foods and beverages containing nanoparticles to ensure consumers have transparency and informed choices. These requirements are crucial, as they allow consumers to make decisions based on the knowledge of the presence of nanomaterials in products (Regulation EC No. 1333/2008, 2008 [107,108]). For example, the European Food Safety Authority (EFSA) provides comprehensive guidelines for the safety assessment of nanoparticles in food and food packaging, emphasizing the need for rigorous evaluations of toxicity, exposure levels, and potential health effects (EFSA, 2018 [108,109]). These assessments include a thorough investigation of the nanoparticles’ potential to migrate from packaging into food products, as well as any possible long-term health effects.

At the international level, in order to promote global trade and ensure consistent standards across borders, efforts are underway to harmonize regulations on nanotechnology in food and beverages. This approach reflects the commitment of regulatory bodies to safeguard public health while fostering innovation in the food industry (ISO, 2019; WHO, 2009 [110]).

While nanoparticles offer exciting possibilities for improving winemaking processes, it is important to carefully consider safety, regulatory compliance, ethical concerns, and the potential long-term effects on wine quality and consumer perception. A thorough risk assessment and compliance with applicable regulations are crucial when integrating nanotechnology into the wine industry [111].

### 5.1. Risk Assessment and Toxicity Concerns

The risk assessment process for nanoparticles in food includes an evaluation of their potential toxicity and the likelihood of exposure to consumers. The unique properties of nanoparticles, such as their ability to penetrate biological barriers and accumulate in tissues, raise concerns about their long-term safety [106]. Regulatory bodies often require data on the physico-chemical properties of nanoparticles, their potential to leach into food or beverages, and their behavior in biological systems. The aim is to ensure that the use of nanomaterials does not pose unforeseen risks to human health.

### 5.2. Harmonizing Global Standards

At the international level, efforts are underway to harmonize regulations on the use of nanotechnology in food and beverages. The World Health Organization (WHO) and the International Organization for Standardization (ISO) are working on guidelines to establish a global consensus on the safety and regulatory assessment of nanomaterials in food (ISO, 2019 [110]; WHO, 2009 [74]). This harmonization is essential to promote global trade, reduce regulatory burdens, and ensure that safety standards are consistent across borders.

### 5.3. The Future of Nanoparticles in Winemaking

As the wine industry explores the potential benefits of nanoparticles, including their use in improving wine quality, enhancing filtration processes, and packaging, it is important to continue to address safety, regulatory compliance, and consumer concerns. Sustainability is becoming a cornerstone of the future of winemaking, driving the adoption of innovative technologies such as nanoparticles. Unlike traditional additives, which can accumulate or require extensive downstream processing, smart surfaces incorporating nanoparticles offer a recyclable and environmentally friendly alternative. These surfaces can act as processing aids, enabling targeted and efficient interactions, such as adsorption or catalysis, without introducing residual chemicals into the wine. This approach minimizes waste and meets the growing consumer demand for sustainable practices and clean-label products, paving the way for a greener future in winemaking. Ongoing research into toxicology and risk assessment of nanoparticles will play a key role in determining how these materials can be safely incorporated into winemaking practices [111]. While nanoparticles offer significant opportunities for innovation in winemaking, careful consideration of their potential risks, along with transparent labeling and compliance with evolving regulations, will be critical to fostering public trust and ensuring the long-term sustainability of the industry.

## 6. Conclusions and Future Perspectives

The future of gold, silver, and magnetic nanoparticles (NPs) in winemaking and viticulture is innovative and promising, with the ability to revolutionize various stages of the winemaking process, from grape cultivation to fermentation, aging, and preservation. These nanoparticles offer unique properties, such as high surface area, enhanced reactivity, and specific interaction capabilities, which can improve wine quality, sustainability, and efficiency. Gold, silver, and magnetic nanoparticles in sensing applications offer a versatile and comprehensive approach to wine analysis and quality control. Nanoparticles are a valuable tool for improving wine production, ensuring wine safety, and exploring the complex world of wine chemistry and flavor due to their unique properties, selectivity, and rapid detection capabilities. Since their use is constantly evolving, they offer new opportunities to advance the art and science of winemaking by enabling innovations in wine production and quality control. In addition, nanoparticles offer a versatile and effective approach to removing or reducing specific compounds from wine. They can selectively interact with and remove undesirable substances while retaining the desired components of the wine. Gold nanoparticles have antimicrobial properties that could help control microbial growth during fermentation or storage, reducing the need for chemical preservatives. Their interaction with the phenolic compounds that contribute to wine flavor could potentially enhance or accelerate the aging process, offering winemakers a faster, more controlled way to develop complex flavors traditionally achieved only through long aging. Moreover, gold nanoparticles can be integrated into wine packaging for real-time monitoring, employing gold-based sensors to detect spoilage or contamination.

Silver nanoparticles are recognized for a broad spectrum of antimicrobial and antifungal properties. In winemaking, they could be applied to prevent microbial contamination during fermentation and aging, potentially reducing reliance on sulfites and other preservatives. Silver nanoparticles could also extend wine’s shelf life by preventing bacterial and fungal growth in packaging. However, they must be used responsibly and in accordance with the relevant regulations. With further research, AgNPs may become a valuable addition to modern winemaking applications.

Magnetic nanoparticles offer exciting opportunities in viticulture and enology. They can be used to modify yeast or bacterial strains to improve fermentation efficiency, achieve desired flavor profiles, or control alcohol content. They can also be used to deliver additives such as tannins or antioxidants more precisely, giving winemakers greater control over flavor and aging characteristics. In addition, the nanoparticles can improve the clarification process, making it easier to remove post-fermentation yeast sediment and reduce the risk of fermentation plant fouling.

While these nanoparticles have great potential, scaling up production at a reasonable cost will be essential for their integration into winemaking processes. Research into the long-term implications of their use is also essential. While recognizing the promise of these developments, we must also be aware of the challenges and regulatory considerations associated with the use of nanoparticles in enology. As nanotechnology becomes increasingly associated with winemaking, ethical and safety issues will also need to be addressed.

## Figures and Tables

**Table 1:** Properties, functions, and applications of nanoparticles as antibacterial and antifungal agents in food and wine.

Function/Properties	Silver Nanoparticles	Gold Nanoparticles	Magnetic Nanoparticles
Antibacterial and Antifungal Activity	Broad-spectrum activity: Effective against Gram-positive and Gram-negative bacteria and fungi commonly found in food and wine (e.g., *E. coli*, *Brettanomyces*) Superior antimicrobial power compared to silver ions Prevents or disrupts biofilm formation, useful for wine spoilage organisms (e.g., *lactic acid bacteria*, *Acetobacter*) [75,76,77,78]	Broad-spectrum activity: Effective against both bacterial and fungal pathogens, though less potent than AgNPs Safer and more colloidally stable than AgNPs, with potential for longer shelf life in food and wine Low toxicity to human cells, making them safe for wine and food applications [79,80,81,82,83]	Effective against various pathogens in food and wine, with enhanced activity through surface modifications and ion release (Fe^2+^, Fe^3+^) Antibacterial properties are often enhanced with functionalization for food and wine spoilage prevention Can inhibit biofilm formation, improving antimicrobial effects in wine preservation [84,85]
Toxicity	Can be toxic at high concentrations, requiring careful control, especially in wine due to potential ion leaching [75,76,86]	Generally considered biocompatible, with low cytotoxicity in both food and wine [81,87,88]	Low toxicity but depends on particle size and surface modification; some concerns with agglomeration in wine [89,90]
Food and Wine Applications	Used in food packaging, preservation, and surface coatings to prevent microbial contamination in both food and wine [2,91,92,93,94]	Applied in wine preservation and food packaging and as part of biosensors for spoilage detection (e.g., sensors for *Saccharomyces cerevisiae*) [2,80,82,83,93,94]	Used in food and wine packaging, coatings, and sensors; useful for targeted antimicrobial delivery in wine filtration [2,93,94]
Mechanism of Action	Disruption of bacterial and fungal cell membranes, release of silver ions, inhibition of microbial DNA replication	Interaction with cell membranes, release of ions, oxidative stress induction, beneficial in preventing spoilage	Magnetic properties allow for targeting specific pathogens in food and wine, controlled release of antimicrobial agents
Biofilm Interference	Can prevent or disrupt biofilm formation, especially in wine spoilage bacteria and fungi, enhancing preservation [84,95,96]	Less effective against biofilm formation than AgNPs but still useful in wine spoilage inhibition [96]	Can disrupt biofilm formation, improving the efficacy of antibiotics and antimicrobial agents in both food and wine [84]
Advantages	Strong and broad-spectrum antimicrobial activity, long-lasting effect in both food and wine storage	Biocompatibility, ease of functionalization, and suitability for use in food and wine applications such as spoilage detection	Magnetic properties allow for easy separation, targeting, and controlled release of antimicrobial agents in food and wine systems
Challenges	Potential environmental impact, issues with AgNP stability and leaching in wine; possible off-flavors or color changes	Limited intrinsic antimicrobial properties in wine, higher cost compared to AgNPs, need for functionalization	Requires careful functionalization for food and wine applications, risk of agglomeration, possible interference with wine flavor

## Data Availability

Data are contained within the article.
